# Is rotavirus still a major cause for diarrheal illness in hospitalized pediatric patients after rotavirus vaccine introduction in the Saudi national immunization program?: Erratum

**DOI:** 10.1097/MD.0000000000007314

**Published:** 2017-06-16

In the article, “Is rotavirus still a major cause for diarrheal illness in hospitalized pediatric patients after rotavirus vaccine introduction in the Saudi national immunization program?”,^[1]^ which appeared in Volume 96, Issue 15 of *Medicine* , two section headings appeared incorrectly. “3.1.1. Residence” should have appeared as “3.1.3. Residence.” “3.1.1. Socioeconomic risk factors” should have appeared as “3.1.4. Socioeconomic risk factors.”

## References

[1] Hegazi M-A Sayed M-H Sindi H-H Is rotavirus still a major cause for diarrheal illness in hospitalized pediatric patients after rotavirus vaccine introduction in the Saudi national immunization program? *Medicine*. 2017 96:e6574. 28403085 10.1097/MD.0000000000006574 PMC5403082

